# Detection of Focal and Non-Focal Electroencephalogram Signals Using Fast Walsh-Hadamard Transform and Artificial Neural Network

**DOI:** 10.3390/s20174952

**Published:** 2020-09-01

**Authors:** Prasanna J., M. S. P. Subathra, Mazin Abed Mohammed, Mashael S. Maashi, Begonya Garcia-Zapirain, N. J. Sairamya, S. Thomas George

**Affiliations:** 1 Department of Electronics and Communication Engineering, Karunya Institute of Technology and Sciences, Tamil Nadu 641114, India; prasanna17@karunya.edu.in (P.J.); sairamyanj@karunya.edu.in (N.J.S.); 2 Department of Electrical and Electronics Engineering, Karunya Institute of Technology and Sciences, Tamil Nadu 641114, India; subathra@karunya.edu; 3 College of Computer Science and Information Technology, University of Anbar, 11, Ramadi, Anbar, Iraq; mazinalshujeary@uoanbar.edu.iq; 4 Software Engineering Department, College of Computer and Information Sciences, King Saud University, Riyadh 11451, Saudi Arabia; mmaashi@ksu.edu.sa; 5 Evida Lab, University of Deusto, Avda/Univesidades 24, 48007 Bilbao, Spain; mbgarciazapi@deusto.es; 6 Department of Biomedical Engineering, Karunya Institute of Technology and Sciences, Tamil Nadu 641114, India

**Keywords:** fast Walsh–Hadamard transform, feature extraction, entropy, classification, artificial neural network

## Abstract

The discrimination of non-focal class (NFC) and focal class (FC), is vital in localizing the epileptogenic zone (EZ) during neurosurgery. In the conventional diagnosis method, the neurologist has to visually examine the long hour electroencephalogram (EEG) signals, which consumes time and is prone to error. Hence, in this present work, automated diagnosis of FC EEG signals from NFC EEG signals is developed using the Fast Walsh–Hadamard Transform (FWHT) method, entropies, and artificial neural network (ANN). The FWHT analyzes the EEG signals in the frequency domain and decomposes it into the Hadamard coefficients. Five different nonlinear features, namely approximate entropy (ApEn), log-energy entropy (LogEn), fuzzy entropy (FuzzyEn), sample entropy (SampEn), and permutation entropy (PermEn) are extracted from the decomposed Hadamard coefficients. The extracted features detail the nonlinearity in the NFC and the FC EEG signals. The judicious entropy features are supplied to the ANN classifier, with a 10-fold cross-validation method to classify the NFC and FC classes. Two publicly available datasets such as the University of Bonn and Bern-Barcelona dataset are used to evaluate the proposed approach. A maximum sensitivity of 99.70%, the accuracy of 99.50%, and specificity of 99.30% with the 3750 pairs of NFC and FC signal are achieved using the Bern-Barcelona dataset, while the accuracy of 92.80%, the sensitivity of 91%, and specificity of 94.60% is achieved using University of Bonn dataset. Compared to the existing technique, the proposed approach attained a maximum classification performance in both the dataset.

## 1. Introduction

Epilepsy is a nervous system syndrome originated by the frequent occurrence of the seizures [1] owing to the sudden abnormal flow of electrical activity in the central nervous system which results in unconsciousness, disability in body movement [2], and also sudden unexpected death in epilepsy (SUDEP) that occurs one in 1000 adults and one in 4500 children each year. According to the International League Against Epilepsy (ILAE), drug-resistant epilepsy is referred to as the epileptic seizures which are unresponsive to the antiepileptic drugs and which results in the treatment through surgery. About more than 60% of the drug-resistant patients are affected with focal epilepsy, which affects the particular section of the neural system, whereas 20% of drug-resistant patients have generalized epilepsy; all parts of the brain are influenced [3]. The estimation of the epileptogenic zone (EZ) before the surgical procedure is a primary requirement for the neurosurgeons.

The electroencephalogram (EEG) is non-invasive clinical equipment to screen the characteristics of the encephalon activity [4] by attaching the electrodes on the scalp over a certain duration known as scalp EEG whereas implanting electrodes in the cerebrum before the surgical treatment is known as intracranial EEG. The conventional EEG-based approach for epileptic diagnosis and estimation of EZ needs tiresome manual inspection of EEG signals done by eminently qualified neurologists [5], hence an increasing demand to automate it is on the rise. In recent years, to assist the neurologists in identifying the EZ, many computer-aided automated diagnosis tools have been developed using signal processing techniques and machine learning algorithms.

In literature, various machine learning [6] methodologies have been proposed [7] for health care problems [8,9] to provide a solution. Various entropy measures with the combination of classifiers were introduced to provide the classification of non-focal class (NFC) and focal class (FC) EEG signals in the Bern-Barcelona (BB) dataset [10]. In [11], 15 levels of flexible analytic wavelet transform (FAWT) were used for the EEG signal decomposition into different sub-bands, and the entropy measures were computed from every sub-band to classify the signals, which attained 94.41% classification accuracy with least-square support vector machine (LS-SVM) classifier. In [12], authors computed the sample entropy (SampEn), Reyni’s entropy (RE), and approximate entropy (ApEn) from EEG signals, and fed it into Non-Nested Generalized Exemplars classifier (NNge) for discriminating the NFC and FC, which achieved an accuracy of 98%. In [13], authors achieved an accuracy of 94.5% using LS-SVM classifier, with the extracted entropy features from the decomposed sub-bands using the orthogonal wavelet filter banks, which are based on the time-frequency domain. In [14], the neighborhood component analysis (NCA) with support vector machine (SVM) attained the sensitivity of 97.6%, the accuracy of 96.1%, and specificity of 94.4% in distinguishing the NFC and FC EEG signals.

In [15], empirical mode decomposition (EMD) was employed for the decomposition of the signals into various intrinsic mode functions (IMF), and log energy (LogEn) entropy feature was extracted from each IMF. An accuracy of 89.4% was achieved using the k-nearest neighbor (k-NN) classifier with the computed features. In [16], tunable-Q wavelet transform (TQWT) was used to classify the NFC and FC with the LS-SVM classifier. In [17], four different classifiers were used with the differential entropy feature vector for classification of NFC and FC. Among these, the random forest (RF) classifier attained specificity of 95%, accuracy of 78.5%, and sensitivity of 95%. In [18], discrete wavelet transform (DWT) with level six and feature ranking methods was used for discriminating the NFC and FC. In [19], the TQWT was employed to decompose the EEG signals, and the LS-SVM classifier with the entropy features provided better discrimination of NFC and FC EEG signals. In [20], the 50 pairs of NFC and 50 pairs of FC EEG signals were used to evaluate the performance of entropy measures in discrimination of NFC and FC. In [21], the empirical wavelet transform (EWT) was employed to analyze the NFC and FC EEG signals. In [22], EMD with entropy features and LS-SVM was employed to discriminate 3750 pairs of NFC and FC EEG signals. In [23], the author used DWT to analyze the EEG signals and various coefficients decomposed, and from each coefficient, the statistical data as features were extracted. The University of Bonn dataset and the BB dataset were used to evaluate the DWT with the statistical feature approach in the classification of NFC and FC EEG signals, with SVM classifier. For the University of Bonn database, an accuracy of 88%, and for the BB dataset, an accuracy of 83.74% was achieved. In [24], multiple features and SVM classifiers were employed for the classification of NFC and FC. This method achieved an accuracy of 92.15%, sensitivity of 94.56%, and specificity of 89.74%. In [25], delay PermEn method and SVM classification were performed to localize the EZ. The adaptive neuro-fuzzy inference system (ANFIS) classifier was used to discriminate the NFC and FC cases using the extracted features, which achieved 100% specificity, 99% accuracy, and 98% sensitivity [26]. In [27], Filter banks were applied to obtain different frequency bands from the signals, and they attained an accuracy of 89.7% for 50 pairs and 89.52% for 750 pairs of NFC and FC EEG signals with LS-SVM classifier, respectively. In [28], the extracted entropy features were fed into the LS-SVM classifier to characterize the FC signal and achieved an accuracy of 82%. In [29], FAWT was developed to decompose the EEG signal into several sub-bands, and fractal dimension features were extracted. The obtained features were classified by a classifier. In [30], a complex wavelet transform (CWT) was applied, and statistical features were supplied into the ANFIS classifier to classify NFC and FC. In [31], DWT was used for the extraction of the features where the optimum features were selected from the extracted statistical features by engaging the genetic algorithm (GA). The comparative study of three different classifiers in distinguishing the NFC and FC was studied.

In [32], an unsupervised methodology of NFC and FC classification performed by autoregressive (AR) modeling, the non-parametric statistical test was performed to provide efficient classification. In [33], energy measures and entropies were analyzed from the differential operation of the EEG signal, those features were fed into SVM for classification, and they got an accuracy of 88.14%. In [34], they used a bivariate EMD to classify NFC and FC signals. Entropy features were extracted, and those were given to the SVM and achieved 86.89% of accuracy. In [35], they used three distinct deep neural networks such as LeNet, AlexNet, and GooLeNet to discriminate NFC and FC EEG signals, and their method achieves a classification accuracy of 100%. In [36], long short-term memory (LSTM) algorithm was applied for the classification, with different k-folds. Based on several experiments, the 10-fold validation was chosen to be the best. In [37], synchro squeezing transform and deep convolutional neural network were utilized, and it provides better classification performance. In [38], a convolutional neural network was applied on the scalp EEG signals to outperform the classification. In [39], the authors have proposed the supervised epileptic seizure detection algorithm to analyze long term EEG signals for reducing the computational complexity. In [40], EEG rhythm-based Taylor Fourier filter bank was designed, and two classifiers, namely, K-NN and SVM classifiers were used engaged, which achieved an accuracy of 94.88%. In [41], FAWT decomposition method with entropy features was classified by the SVM classifier, which achieved better accuracy. In [42], variational mode decomposition (VMD) was performed on the EEG signals in the DWT domain, and the computed entropy features classified by ensemble stacking provided 95.2% accuracy. In [43], EMD method was employed to investigate signals justified as IMFS. The features were extracted from the IMFs via phase space reconstruction and classified by the neural networks. In [44], the third-order cumulant function was proposed for the discrimination of EEG signals with the help of the SVM classifier.

Although the various methods in the literature have provided an efficient classification performance, still their respective performance has to be improved. Hence, in this work, the FWHT technique is used to analyze the NFC and FC signals, and five individual entropy measures are extracted from the decomposed EEG signals. ANN classifier is utilized to classify the extracted features for evaluating the performance of the proposed approach in discrimination of NFC and FC EEG signals. For this work, the two datasets, namely the University of Bonn and BB dataset, are used. The performance is evaluated, and the results with the state-of-art techniques are compared. The proposed method attained the highest classification performance in both datasets.

## 2. Materials and Methods

### 2.1. Dataset Used

In this paper, the two publicly available EEG databases for the automatic classification of NFC and FC EEG signals are used.

#### 2.1.1. University of Bonn Dataset

**Figure 1:**
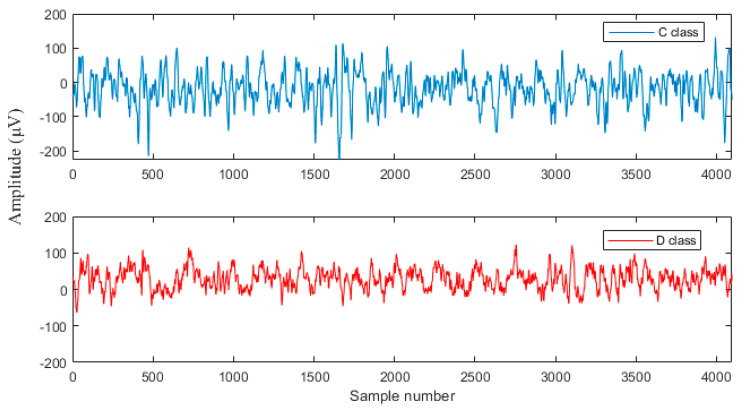
Sample of University of Bonn dataset.

The University of Bonn EEG dataset was used to analyze the proposed work. The dataset consists of 500 EEG recorded files with five classes (A, B, C, D, and E), every single containing single-channel EEG sectors with 100 signals [45]. A and B classes are recordings of the healthy person during eyes open and close. C and D classes are recordings of the epileptic subjects during the interictal stage during the non-existence of seizures. E class is recordings of the epileptic subject during seizure activity. The removal of artifacts is done by visual examination. This work mainly focuses on the classification of NFC and FC EEG signals. Hence, the classes C and D were alone used in this work. The C and D groups consist of EEG signals of duration 23.6 s, which was measured from the focal epileptic subjects. The EEG signals measured at EZ using the depth electrodes are in class D, and class C consists of EEG signals measured from the hippocampal formation of the opposite hemisphere of the brain. Each EEG record consists of 4096 samples, and the sampling frequency is 173.61 Hz. The total time lengths of the signals in the dataset are 23.6 s [45]. The samples of C and D EEG signals are shown in Figure 1.

#### 2.1.2. Bern-Barcelona Dataset

An open-access dataset BB was engaged in conducting all the experimentations in this present work. The dataset consists of intracranial EEG recordings of two classes, namely NFC and FC. Each class contains 3750 pairs of EEG recordings represented by x and y from five epileptic subjects [46] who have experienced the temporal lobe epilepsy, where all the subjects had a surgical treatment result. In FC recordings, x is recorded to discern the 1st ictal signal from the channels, and y is denoted as neighboring channels. In NFC, x and y are the records of the remaining neighboring channels. Three subjects were cured completely from seizures, whereas two subjects had the sensation of the seizures. FC signals were obtained from the epileptic region, whereas NFC signals were collected from the other region of the brain.

**Figure 2:**
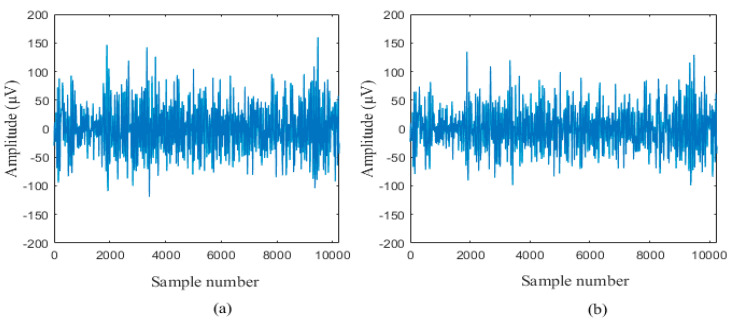
A sample of non-focal class (NFC) electroencephalogram (EEG) signal (**a**) x channel and (**b**) y channel.

**Figure 3:**
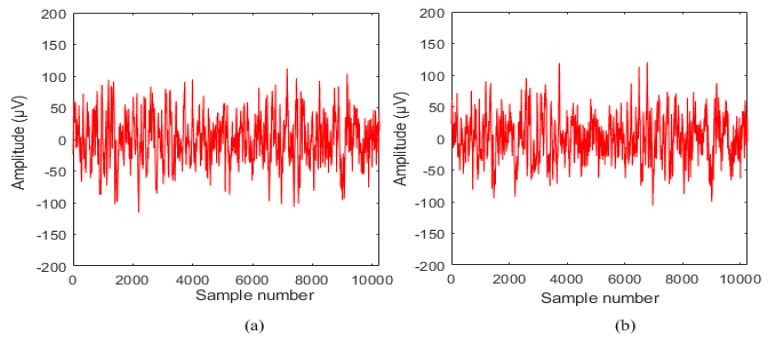
A sample of focal class (FC) EEG signal (**a**) x channel and (**b**) y channel.

Each EEG recordings consist of 10,240 samples with a sampling rate of 512 Hz [22]. These EEG records were preprocessed using the bandpass filter to remove artifacts. The time duration of each EEG recording is 20 s. The entire 3750 pairs of NFC and FC recordings in the BB dataset were utilized in this study. The sample of NFC and FC signals are shown in Figure 2 and Figure 3.

### 2.2. Fast Walsh Hadamard Transform

**Figure 4:**
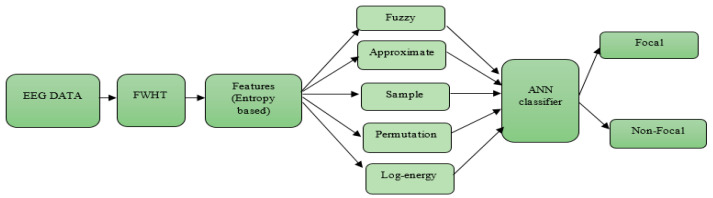
Block diagram of the proposed work.

In this paper, a technique based on the frequency domain utilized for the classification of NFC and FC EEG signals is proposed. The block diagram of the proposed method is given in Figure 4. FWHT was used to extract the discriminating features from the EEG signal [47]. FWHT is an efficient technique to estimate the Walsh–Hadamard Transform (WHT), which involves the conversion of time-domain signals into frequency domain [48].

FWHT decays the signal into a set of orthogonal, rectangular waveforms called Walsh functions with the values of +1 or −1 [49]. The unique sequence value will be assigned with each Walsh function. The execution of the FWHT algorithm is comprehended by the addition and subtraction function. FWHT has the competency to identify the signals more precisely, that consist of a sharp disruption in the signal. FWHT decomposes the signal samples with a length of $2^{n}$ into $2^{n}$ coefficients. FWHT returns the coefficient of the Discrete Walsh–Hadamard Transform (DWHT) of the signal. The FWHT of the data samples of *x*(*n*) and n=1,2, …… N  is given as follows [50]

(1) $$\;\;\;\;\;\;\;\;\;\;\;\;X_{w}\left(k\right)=\overset{N}{\underset{n=1}{\sum }}x\left(n\right)\;W_{n}\;\;\;\;\;k=1,2,\;\ldots \ldots \;N\;\;\;\;\;\;\;$$

where *N* indicates the total samples number and $W_{n}\;$ is the Walsh function (Walsh matrix) which is given by the following equation [51]

(2) $$\;\;\;\;W_{n}=\frac{1}{2^{\frac{n}{2}}}\left(\begin{array}{cc} W_{n-1} & W_{n-1}\\ W_{n-1} & -W_{n-1} \end{array}\right)\;$$

In the 1 × 1 matrix the value of $W_{0}$ is equal to 1. Therefore, $W_{1}$ and $W_{2}$ can be written as

(3) $$\;\;\;W_{1}=\frac{1}{\sqrt{2}}\left[\begin{array}{cc} 1 & 1\\ 1 & -1 \end{array}\right]\;$$

(4) $$W_{2}=\frac{1}{2}\left[\begin{array}{cc} \begin{array}{cc} 1 & 1\\ 1 & -1 \end{array} & \begin{array}{cc} 1 & 1\\ 1 & -1 \end{array}\\ \begin{array}{cc} 1 & 1\\ 1 & -1 \end{array} & \begin{array}{cc} -1 & -1\\ -1 & 1 \end{array} \end{array}\right]\;$$

The Walsh function can also be written as

(5) $$\;\;\;\;\;\;W_{n}\;\;=\overset{m}{\underset{i=1}{\prod }}{\left(-1\right)}^{n_{i}k_{m-i}}\;\;\;$$

The FWHT is a product of a data sequence whose length is 1 × N and the Walsh matrix with a length of N × N [50].

**Figure 5:**
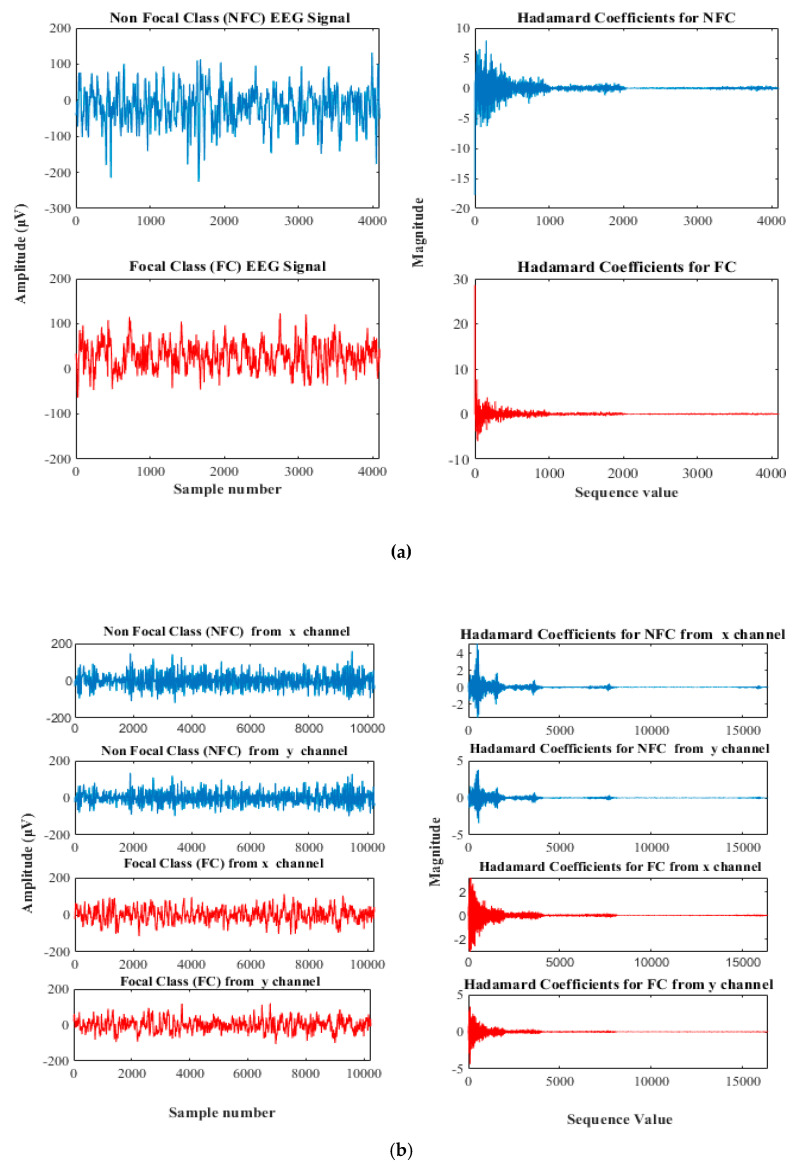
The plot of EEG signal and its Hadamard coefficient for (**a**) University of Bonn and (**b**) Bern-Barcelona Dataset.

The main advantage of this transform is it requires less storage space to store decomposed coefficients, and signal reconstruction is faster. FWHT works on the signals with length equal to a power of 2. If the length is less than the power of 2, its length is padded with zeros to the next greater power of 2 before processing [52]. In this work, the FWHT was applied to analyze the EEG signal from the University of Bonn database, of sample size 4096, which is two to the power of 12 ($2^{12}$). Hence, it is decomposed to 4096 Hadamard coefficients. Whereas, in the BB data set, 10,240 samples are available. Since it has value less than the power of two (10,240 > $2^{13}$) it is padded with zeroes till the next greater power of two so that 16,384 ($2^{14}$) Hadamard coefficients are decomposed and shown in Figure 5.

### 2.3. Feature Extraction

Feature extraction is an essential process for event classification of signals and images in the medical field for clinical diagnosis [53]. In this study, five nonlinear entropy-based discriminating features from the decomposed Hadamard coefficient of EEG signal were extracted. The entropy features compute the randomness of the time sequence of the EEG signals algebraically. These measures reflect the information of inspected anarchy within the intracortical area [54]. The extracted features are given as follows:

#### 2.3.1. Approximate Entropy (ApEn)

ApEn computes the stochasticity and consistency in the manifold dimension. It is a scale-invariant which relates the resemblance of the samples in the EEG [54]. The logarithmic probability that the signal with N sample points reiterates itself within the lenience of p for k points and for next m + 1 points is expressed in ApEn. For a given time sequence y(i) of length N, length *N – k + 1* vectors Y(1), Y(2),…,Y(*N – k + 1*) are constructed. The ApEn is

(6) $$ApEn\left(k,p,N\right)=\;\phi^{k}\left(p\right)-\phi^{k+1}\left(p\right)$$

where

(7) $$\;\;\;\;\;\phi^{k}\left(p\right)=\;\frac{1}{\left(N-k+1\right)}\underset{i}{\sum }\ln\left(C_{i}^{d}\left(p\right)\right)\;\;\;$$

where, $C_{i}^{d}$ is denoted as correlation integral signifying the probability of a vector Y(i) remaining alike to Y(j) in lenience limit *p*. It is widely engaged in the miscellaneous zone of biomedical signal processing.

#### 2.3.2. Sample Entropy (SampEn)

SampEn is used to compute the consistency in the signal efficiently than ApEn [55], and it is a revised form of ApEn, beneficial for complexity reduction in the EEG signal. It is not reliant on the length of the time cycles [12]. The greater SampEn value suggests that the signal is predicted less effectively, and a lower SampEn predicts the signal more effectively [54]. In SampEn, the bias instigated by the self matches is prevented from extemporizing the function. For a given signal y(i), the sample entropy is given as follows

(8) $$SampEn\left(m,r,N\right)=-\ln\left(\frac{A^{m}\left(r\right)}{B^{m}\left(r\right)}\right)$$

where the parameters are defined as

(9) $$\;\;\;\;B^{m}\left(r\right)=\;\frac{1}{\left(N-m\right)}\;\overset{N-m}{\underset{i=1}{\sum }}C_{i}^{m}\;\left(r\right)\;\;\;\;\&\;\;\;\;A^{m}\left(r\right)=\;\frac{1}{\left(N-m\right)}\;\overset{N-m}{\underset{i=1}{\sum }}C_{i}^{m+1}\;\left(r\right)\;\;\;$$

(10) $$C_{i}^{m}\;\left(r\right)=\;\frac{1}{\left(N-m\right)}\;C_{i},\;\;\;\;\;\;\;\;\;i=1,2,\ldots .,\;N-m$$

where, $C_{i}$ is the sum, L[Y(i),Y(j)] ≤ *r*, without the self matches. The parameter L[Y(i),Y(j)] is the interval between Y(i) and Y(j) and can be written as

(11) $$L\left[Y\left(i\right),Y\left(j\right)\right]=\;\underset{1,2,\ldots .,m}{\max}\left(\left|y\left(i+k-1\right)-y\left(j+k-1\right)\right|\right)\;\;\;$$

#### 2.3.3. Permutation Entropy (PermEn)

PermEn is employed to estimate the difficulty in the time sequence by comparing the neighbor time-series signals [56]. It is a non-stationary time series approach that provides efficient and robust and quick results [54]. The relationship between the distinct number of equidistant values for past and present esteem are labeled by mapping onto a symbolic sequence from the continuous-time sequence record. *y*(*t*) for *t* = 1, 2, … is embedded into a *k* dimensional space to accomplish the mapping. The time-series data can be as follows [57]

(12) Y(t)=[y(t),y(t+τ),….,y(t+kτ)]

where, *k* corresponds to the embedding measurement, and *τ* is the time gap. For the *k* embedding dimension, the conceivable permutations will be *k*!. The embedded time vectors *X*(*t*) will be given by a *j* symbol sequence, and *p_j_* represents a probability distribution for each symbol. *H*_PermEn_, of a specified time sequence, *x*(*t*), is defined as follows [57]

(13) $$H_{\mathrm{PermEn}}\left(k\right)=-\frac{1}{\ln\left(k!\right)}\overset{J}{\underset{j=1}{\sum }}p_{j}\;\ln\left(p_{j}\right)$$

where the number of dissimilar symbols is *J* for a specified embedding dimension that is (*J* ≤ *k*!). The normalization factor is written as $\frac{1}{\ln\left(k!\right)}$ therefore 0 ≤$\;H_{\mathrm{PermEn}}/\frac{1}{\ln\left(k!\right)}\;$≤1.

#### 2.3.4. Fuzzy Entropy (FuzzyEn)

FuzzyEn signifies the information of consistency, and it is implemented from the fuzzy sets [58]. FuzzyEn measure on the EEG signal has a specific function, which is known as the membership function, with a value that remains from 0 to 1 [59]. FuzzyEn is a continuous function which is used to estimate resemblance in the time sequence of the signal [60]. The time sequence signal of *y*(*i*) with N signifies the sample number and *k* represents the length, the vector sequences are considered as

(14) $$Y_{i}^{k}=\{y\left(i\right),y\left(i+1\right),$$

where, $Y_{i}^{k}$ denotes successive values, instigated with the *k^th^* point and comprehensive by baseline removal.

(15) $$y0\left(i\right)=\frac{1}{k}\;\overset{k-1}{\underset{j=0}{\sum }}y\left(i+j\right)$$

The fuzzy function written in exponential form is given as,

(16) $$\mu \left(d_{ij}^{k},n,p\right)=\exp(-\left(d_{ij}^{k}{)}^{n}/p\right)$$

where $d_{ij}^{k}$ is the absolute distance of the points.

The function $\phi^{k}$ for $Y_{i}^{k}$ and $\phi^{k+1}$ for $Y_{i}^{k+1}$ is given as

(17) $$\;\;\;\phi^{k}\left(n,p\right)=\;\frac{1}{N-k}\overset{N-k}{\underset{i=1}{\sum }}(\frac{1}{N-k-1}\overset{N-k}{\underset{j=1\;j\neq i}{\sum }}\mu \left(d_{ij}^{k},n,p\right))\;\;$$

(18) $$\;\phi^{k+1}\left(n,p\right)=\;\frac{1}{N-k}\overset{N-k}{\underset{i=1}{\sum }}(\frac{1}{N-k-1}\overset{N-k}{\underset{j=1\;j\neq i}{\sum }}\mu \left(d_{ij}^{k+1},n,p\right))\;$$

For the finite dataset, the factor of FuzzyEn (*k*,*n*,*p*) of the negative natural logarithm order of the deviancy of $\phi^{k}$ and $\phi^{k+1}$ [58]

(19) $$\mathrm{FuzzyEn}\;\left(k,n,p\right)=\ln\phi^{k}\left(n,p\right)-\ln\phi^{k+1}\left(n,p\right)$$

#### 2.3.5. Log-Energy Entropy (LogEn)

LogEn is employed to compute the amount of contribution of signals. Mathematically, LogEn is formulated as

(20) $$\mathrm{LogEn}=\;\overset{N}{\underset{i=1}{\sum }}\log(y_{i}^{2})$$

where, *N* corresponds to the total interval of the signal, and *y_i_* is the *i^th^* signal samples. In [15,61], the authors attained high classification accuracy by LogEn feature.

### 2.4. Artificial Neural Network (ANN) Classifier

**Figure 6:**
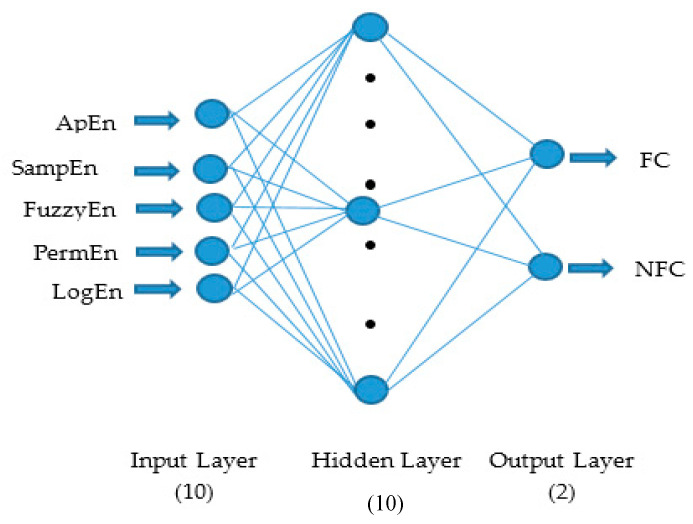
Artificial neural network.

ANN comprises the output layer, input layer, and one or more hidden layer (middle layer) with a more number of processing nodes or artificial neurons connected with each other shown in Figure 6. The connection between two neurons controlled by weights, which can be adjustable so as to increase the accuracy of the system [62]. ANN is a biologically inspired model that classifies a single layer and multiple layer architecture where the feed-forward multilayered neural network is communally used for physical modeling [63]. ANN follows the supervised learning methodology, and also it overwhelmed the hitch due to the nonlinearly separable problem in the single-layer perceptron.

ANN learning is realized by various training algorithms based on training rules or functions. The training algorithm involves training the neural network to perform its function. In this work, five entropy features per channel, hence a total of 10 nonlinear features, are given as an input to the input layer to perform the discrimination between the NFC and FC signals. A feed-forward multilayered neural network is communally used for physical modeling [63]. The few hidden neurons are not efficient for classification [64]. Hence, the total hidden neurons considered in this study is 10. Levenberg–Marquardt backpropagation (LMBP) training algorithm, which is a class of quasi-newton algorithms, is used to train the neural network. LMBP algorithm is an iterative approach that discovers the minimum function that is an amount of quadrangle of nonlinear function [65].

The classification performance is calculated for individual NFC and FC subjects based on the classification sensitivity, accuracy, positive predictive value, specificity, and negative predictive value which are given below

(21) $$Accuracy=\;\frac{T_{+}+T_{-}}{T_{+}+T_{-}+F_{+}+F_{-}}*100$$

(22) $$Sensitivity=\;\frac{T_{+}}{T_{+}+F_{\_}}*100$$

(23) $$Specificity=\;\frac{T_{N}}{T_{-}+F_{+}}*100$$

(24) $$Positive\;predictive\;value=\;\frac{T_{+}}{T_{+}+F_{+}}*100\;\;\;\;$$

(25) $$Negative\;predictive\;value=\;\frac{T_{-}}{T_{-}+F_{-}}*100$$

In the above equation $T_{+}$ corresponds to true positive, $T_{-}$ is defined as true negative, $F_{+}$ referred to as false positive, and $F_{-}$ represents false negative.

## 3. Results and Discussion

**Figure 7:**
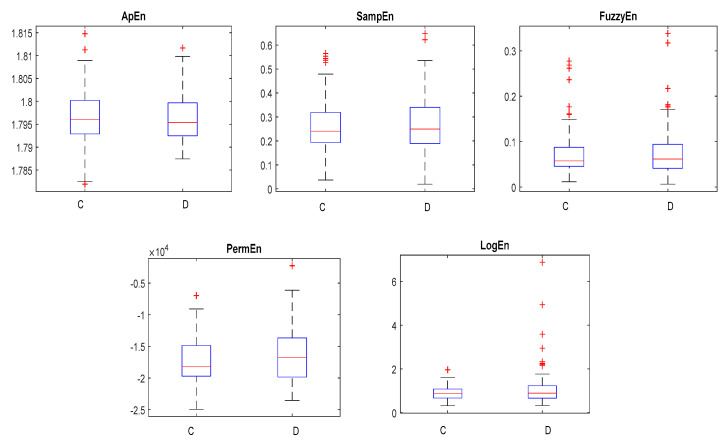
The box plots of the C and D classes in the University of Bonn dataset.

**Figure 8:**
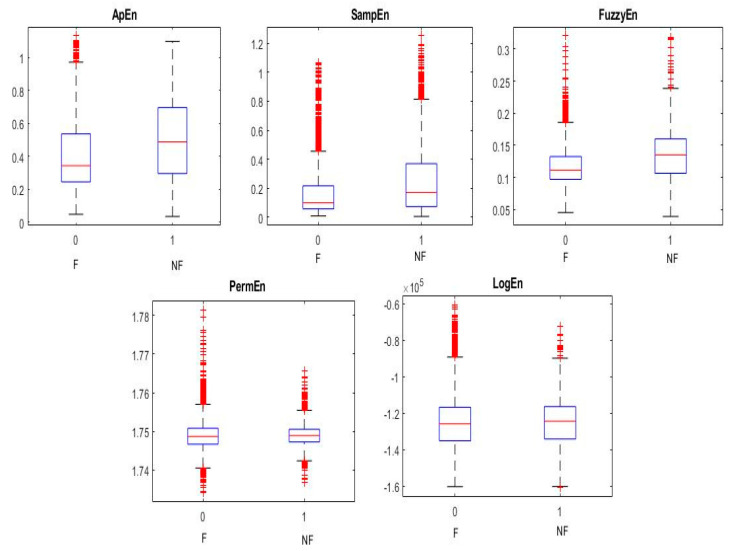
The box plots of the NFC and FC classes in the Bern-Barcelona dataset.

The NFC and FC EEG signals acquired from BB and University of Bonn dataset datasets were employed to investigate the proposed methodology. In this work, the Hadamard coefficients in the individual pairs of the NFC and FC EEG signals were calculated by the effective decomposition method. FWHT transforms the time series signals to the frequency-dependent signals so as to analyze the nonlinear characteristics. In the BB dataset, 3750 pairs of signals and C and D classes of the University of Bonn dataset were taken for the experiment. Judicious features were extracted from the decomposed Hadamard coefficient of EEG signals that were used for the identification of NFC and FC EEG signals. The entropy features provide the characteristics of the nonlinearity existing in the signals to distinguish the variation present in the two classes for the classification. The entropy measures, namely SampEn, FuzzEn, LogEn, PermEn, and ApEn per channel, were extracted. Hence, a total of 10 features from the BB dataset and five features from the University of Bonn dataset were extracted. Student’s *t*-test was conducted on the extracted features of both databases to identify the statistically significant features (*p* < 0.01). The obtained *p*-values for different extracted features were found to be lesser than 0.01. Hence, all the features were fed into the ANN classifier with different combinations listed in Table 1 and Table 2. Ten-fold cross-validation was performed to validate the classification performance. Figure 7 and Figure 8 show the variation of the NFC and FC EEG signals in the University of Bonn and BB data, respectively.

The entropy measures with single features and multiple combinations were given as an input to the ANN classifier to identify the feature set with better classification performance. With LogEn feature the classification of NFC and FC using the University of Bonn database attained an accuracy of 66.90%. While in the BB dataset, when the SampEn feature was given as input to the ANN classifier accuracy of 96.84%, the sensitivity of 100% and specificity of 93.69% were attained. In order to improve the precision of the overall performance, the combination of features such as two features, three features, four features, and five features were done for both the datasets and listed in Table 1 and Table 2. Compared to different combinations with SampEn, an accuracy greater than 98% is seen in the BB dataset, while a maximum accuracy of 99.50% was attained for five feature combinations. It can also be noted that the combination of four features (SampEn, FuzzyEn, PermEn, LogEn), attained an accuracy of 94.43%, which is more or less equal to the accuracy attained by the five feature combination. On the other hand, for the University of Bonn database, the combination of five features attained a maximum accuracy of 92.80%. Hence, based on the results, the five feature combination was chosen as the best feature set for the classification of NFC and FC EEG signals.

The classification performance in terms of accuracy, sensitivity, and specificity for the University of Bonn database is 92.80%, 91%, and 94.60%. Similarly, for the BB dataset, the proposed method provides specificity of 99.36%, accuracy of 99.50%, and the sensitivity of 99.70%.

The classification performance of the proposed method was compared with the existing methods, which are tabulated in Table 3.

Various techniques have been proposed for EEG signal classification for epileptic seizure detection. The FAWT decomposition method [11,41] was employed, with LS-SVM classifier, which attained an accuracy of 94.41% and 94.80%, respectively, for the 3750 pairs of signals. Table 3 shows that many authors examined the entropy as features for classification of FC and NFC; it proves that compared to other features, different entropy features are more discriminate in classifying the FC and NFC classes. In [15,16,18,21], they have only examined the 50 pairs of EEG signals. In [15,22,43], EMD method was proposed for the feature extraction. In [42], VMD-DWT decomposition was performed to detect the epileptic seizures and achieved 95.2% of accuracy. In [36], the deep learning LSTM based features were evaluated for the classification of NFC and FC EEG signals. The proposed approach with the BB dataset attained an accuracy of 99.50%. It can also be noted that compared to the existing techniques with DWT, and WPD [66,67,68] the proposed approach attained an accuracy of 92.80% with University of Bonn dataset. Thus, it can be concluded that the proposed method provides a higher classification performance than all other methods.

**Figure 9:**
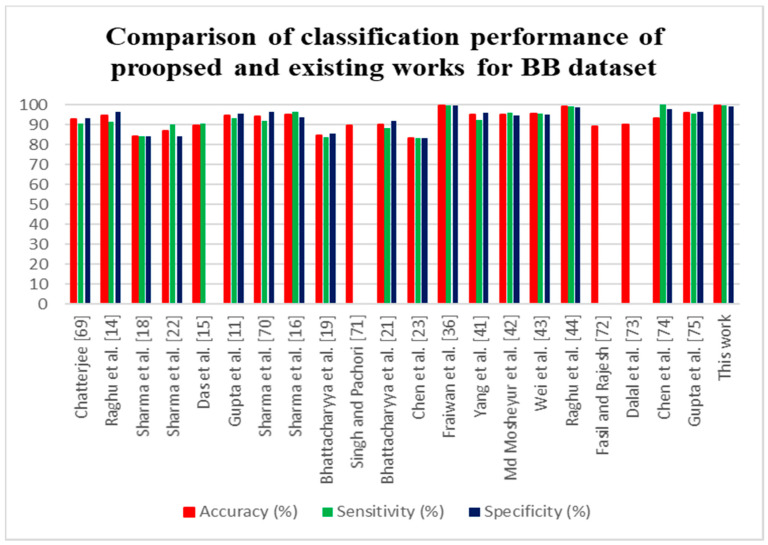
Comparison of classification performance proposed method with existing methods for the BB dataset.

**Figure 10:**
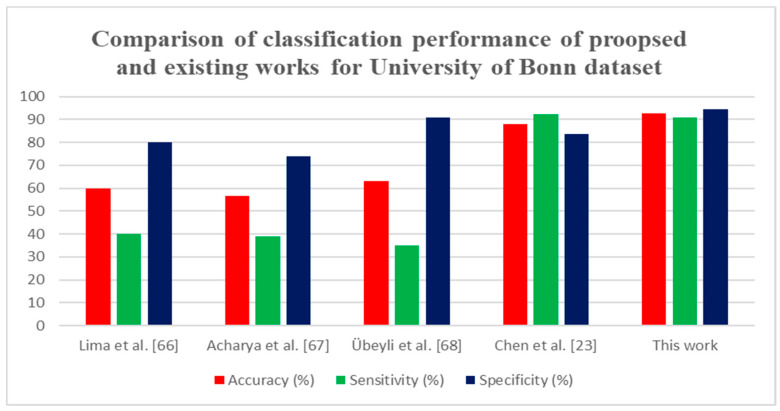
Comparison of classification performance proposed method with existing methods for the University of Bonn dataset.

The comparison of the proposed method with prevailing techniques is presented in Figure 9 and Figure 10 for the BB dataset and University of Bonn dataset. It is also proved that the proposed technique reached maximum accuracy than the already existing techniques. The key benefit of the proposed method is its computational efficiency and constancy. In the future deep learning, approaches will be used to analyze the automatic recognition of epileptic seizures.

## 4. Conclusions

The main aspiration of this present study was to afford an automated diagnosis tool for NFC and FC signals, to assist the neurologist in localization of the EZ for the clinical decision making. Therefore, the feature extraction approach called the FWHT method was used to decompose the EEG signals into different coefficients. Five significant entropy features were extracted from the decomposed coefficients and fed into the ANN classifier, for discrimination of NFC and FC signals. The classification performance of single entropy features and multiple combinations of features were employed to obtain a better classification performance. Simulation performance reveals that the combination of five entropies achieved highest classification accuracy. Maximum accuracy of 92.80% was achieved for the University of Bonn dataset and 99.50% for the BB dataset. Compared to the state-of-art techniques, the proposed approach attained maximum accuracy with reduced computational performance. The performance proposed method exceeds other methods, and it can be used for clinical practice at medical centers. Future research direction comprises the advancement of the proposed method using deep learning approaches and to diagnose other diseases, namely neurodegenerative disorders and mental disorders.

## Figures and Tables

**Table 1:** The classification results using the combination of entropy features for the Bern-Barcelona (BB) dataset.

Feature	Accuracy (%)	Sensitivity (%)	Specificity (%)
**Single feature**			
ApEn	62.89	67.22	58.56
SampEn	96.84	100	93.69
PermEn	57.05	40.90	73.20
FuzzyEn	67.82	73.03	62.62
LogEn	59.89	61.80	57.98
**Two features**			
SampEn, ApEn	97.41	99.14	95.68
SampEn, LogEn	98.80	97.98	99.74
SampEn, PermEn	96.75	99.98	93.53
SampEn, FuzzyEn	96.53	94.33	99
ApEn, LogEn	66.89	68.01	65.77
ApEn, PermEn	63.79	63.57	64
ApEn, FuzzyEn	70.89	74.27	67.46
LogEn, PermEn	61.21	58.82	63.60
LogEn, FuzzyEn	70.83	74.05	67.61
PermEn, FuzzyEn	69.44	75.00	63.88
**Three features**			
SampEn, ApEn, LogEn	98.78	99.29	98.17
SampEn, ApEn, PermEn	98.30	99.47	97.13
SampEn, ApEn, FuzzyEn	98.47	99.15	97.80
SampEn, PermEn, LogEn	98.28	99.86	96.69
SampEn, LogEn, FuzzyEn	98.89	99.76	98.02
SampEn, FuzzyEn, PermEn	98.98	99.78	98.17
ApEn, LogEn, PermEn	67.47	67.34	67.59
ApEn, LogEn, FuzzyEn	74.25	78.10	70.41
LogEn, PermEn, FuzzyEn	71.80	74.56	69.05
PermEn, FuzzyEn, ApEn	71.50	74.84	68.16
**Four features**			
SampEn, ApEn, FuzzyEn, PermEn	98.54	99.27	97.81
SampEn, ApEn, FuzzyEn, LogEn	98.40	99.11	97.70
SampEn, FuzzyEn, PermEn, LogEn	99.43	99.89	98.96
SampEn, LogEn, ApEn, PermEn	98.33	99.12	97.55
ApEn, FuzzyEn, PermEn, LogEn	74.95	78.35	71.54
**Five features**			
SampEn, ApEn, FuzzyEn, LogEn, Perm	99.50	99.70	99.30

**Table 2:** The classification results using the combination of entropy features for the University of Bonn dataset.

Feature	Accuracy (%)	Sensitivity (%)	Specificity (%)
**Single feature**			
ApEn	62.60	42	83.20
SampEn	64.15	56.30	72
PermEn	59.80	62.20	57.40
FuzzyEn	64.85	48.90	80.80
LogEn	66.90	64.70	69.10
**Two features**			
SampEn, ApEn	72.60	64.80	80.40
SampEn, LogEn	79.40	72.60	86.2
SampEn, PermEn	67.35	62.80	71.90
SampEn, FuzzyEn	75.80	66.80	84.80
ApEn, LogEn	79.75	72	87.5
ApEn, PermEn	68.70	56.90	80.50
ApEn, FuzzyEn	78.05	70.60	85.50
LogEn, PermEn	76.45	77.40	75.50
LogEn, FuzzyEn	79.60	71.20	88
PermEn, FuzzyEn	72.85	70.40	75.30
**Three features**			
SampEn, ApEn, LogEn	84.35	79.20	89.50
SampEn, ApEn, PermEn	73.50	70.40	76.60
SampEn, ApEn, FuzzyEn	82.35	77.40	87.30
SampEn, PermEn, LogEn	82.75	77.70	87.80
SampEn, LogEn, FuzzyEn	87.35	86.10	88.60
SampEn, FuzzyEn, PermEn	84.15	78.50	89.80
ApEn, LogEn, PermEn	86.70	81.70	91.70
ApEn, LogEn, FuzzyEn	84.30	80	88.60
LogEn, PermEn, FuzzyEn	84	78.40	89.60
PermEn, FuzzyEn, ApEn	84.25	80.40	88.10
**Four features**			
SampEn, ApEn, FuzzyEn, PermEn	90.25	88	92.50
SampEn, ApEn, FuzzyEn, LogEn	89.55	87.70	91.40
SampEn, FuzzyEn, PermEn, LogEn	88	86.50	89.50
SampEn, LogEn, ApEn, PermEn	89.85	88.70	91
ApEn, FuzzyEn, PermEn, LogEn	90.75	87.70	93.80
**Five features**			
SampEn, ApEn, FuzzyEn, LogEn, Perm	92.80	91	94.60

**Table 3:** Comparison of the performance of the proposed and existing method.

Author Name	Number of Signal Pairs	Methodology	Classifiers	Accuracy	Sensitivity	Specificity
**BB dataset**						
Chatterjee [69]	3750 pairs	Higher order moments in EMD-TKEO domain	SVM	92.65	90.70	93.15
Raghu et al. [14]	3750 pairs	NCA and entropies	LS-SVM	94.5	91.5	96.56
Sharma et al. [18]	50 pairs	DWT and seven different entropies	LS-SVM	84	84	84
Sharma et al. [22]	50 pairs	EM, and six different entropies	LS-SVM	87	90	84
Das et al. [15]	50 pairs	EMD, DWT, and three nonlinear features	k-NN	89.4	90.7	-
Gupta et al. [11]	3750 pairs	FAWT and three different entropies	LS-SVM	94.41	93.25	95.57
Sharma et al. [13]	3750 pairs	Orthogonal wavelet filter banks, entropy measures	LS-SVM	94.25	91.95	96.56
Sharma et al. [16]	3750 pairs	TQWT and three different entropies	LS-SVM	95	96.37	93.47
Bhattacharyya et al. [19]	3750 pairs	TQWT based multivariate sub-band fuzzy entropy	LS-SVM	84.67	83.86	85.46
Singh and Pachori [29]	50 pairs	Fourier rhythms, bandwidth features	LS-SVM	89.7	-	-
Bhattacharyya et al. [21]	50 pairs	EWT, area computed from RPS rhythms	LS-SVM	90	88	92
Chen et al. [23]	3750 pairs	DWT and statistical features	SVM	83.07	83.05	83.09
Fraiwan et al. [36]	3750 pairs	-	LSTM	99.60	99.65	99.55
Yang et al. [41]	3750 pairs	FAWT and entropies	LS-SVM	94.80	92.27	96.10
Md Mosheyur et al. [42]	3750 pairs	VMD-DWT and entropies	Ensemble stacking	95.2	96.1	94.4
Wei et al. [43]	3750 pairs	EMD, IMF based	Neural network	95.37	95.52	95.23
Raghu et al. [44]	3750 pairs	Third order cumulant function	SVM	99	99.33	98.66
Fasil and Rajesh [70]	3750 pairs	Time domain exponential energy	SVM	89	-	-
Dalal et al. [29]	50 pairs	Flexible time-frequency coverage analytic wavelet transform and Fractal dimension	Robust energy-based least square twin support vector machine	90.2	-	-
Chen et al. [71]	50 Pairs	ARMA, EMD, singular values	SVM	93	100	97.9
Gupta et al. [72]	3750 pairs	Fourier–Bessel series expansion based flexible time-frequency coverage wavelet transform, mixture correntropy, exponential energy	LS-SVM	95.85	95.47	96.24
This work	3750 pairs	FWHT + Entropies	ANN	99.50	99.70	99.30
**University of Bonn dataset**						
Lima et al. [66]	200 signals	DWT and statistical features	RVM	60	40	80
Acharya et al. [67]	200 signals	WPD and PCA	GMM	56.50	39	74
Übeyli et al. [68]	200 signals	DWT and statistical features	ANN	63	35	91
Chen et al. [23]	200 signals	DWT and statistical features	SVM	88	92.24	83.76
This work	200 signals	FWHT + Entropies	ANN	92.80	91	94.60
